# ﻿A Neotropical complex of *Ripersiella* species (Hemiptera, Coccomorpha, Rhizoecidae) collected from the nests of *Acropyga* ants (Hymenoptera, Formicidae)

**DOI:** 10.3897/zookeys.1123.90141

**Published:** 2022-09-29

**Authors:** Scott A. Schneider, John S. LaPolla

**Affiliations:** 1 United States Department of Agriculture, Agricultural Research Service, Henry A. Wallace Beltsville Agricultural Research Center, Systematic Entomology Laboratory, 10300 Baltimore Avenue, Beltsville, MD, 20705, USA United States Department of Agriculture, Agricultural Research Service Beltsville United States of America; 2 Department of Biological Sciences, Towson University, 8000 York Road, Towson, MD, 21252, USA Towson University Towson United States of America

**Keywords:** Coccoidea, mutualism, root mealybug, taxonomy, trophobiosis

## Abstract

We describe five new Neotropical species of *Ripersiella* living in association with *Acropyga* ants: *R.campensis***sp. nov.**, *R.illicians***sp. nov.**, *R.montanae***sp. nov.**, *R.pediandensis***sp. nov.**, and *R.telalia***sp. nov.** We also redescribe *R.andensis* and *R.colombiensis* based on type specimens and other collections. Together, these seven species form a morphologically similar group that we informally refer to as the *andensis*-complex of *Ripersiella*. All members of the *andensis*-complex are confirmed or are speculated to be mutualists of *Acropyga* ants. We discuss the implications of these associations and provide an identification key to the Neotropical species of *Ripersiella* that are lacking bitubular cerores, including the new species.

## ﻿Introduction

*Acropyga* Roger ants (Hymenoptera, Formicidae) are obligatory mutualists of scale insects. They primarily associate with root mealybugs from the family Xenococcidae Tang (Hemiptera, Coccomorpha), who are likewise obligate mutualists of *Acropyga* (Williams 1998; LaPolla 2004; Williams 2004; Schneider and LaPolla 2011). The ancient relationship (Blaimer et al. 2016) between *Acropyga* and Xenococcidae has been reinforced through the vertical transmission of associated lineages over generations (LaPolla et al. 2002; LaPolla 2005). However, partner fidelity among *Acropyga* species and their trophobionts has been imperfect and a small proportion of colonies associate with scale species belonging to other groups; most often this involves root mealybugs from the family Rhizoecidae Williams (Williams 1998; Johnson et al. 2001; Tanaka 2016; Caballero et al. 2019; Schneider and LaPolla 2020). These secondary relationships are presumed to be the result of horizontal transmission events (see Page 2003) from an ancestral xenococcid partner to a more recently acquired rhizoecid (or other scale) partner (Schneider and LaPolla 2020).

Some species of root mealybugs from the genus *Ripersiella* Tinsley (Hemiptera, Rhizoecidae) have previously been reported as mutualists of *Acropyga* ants, including *R.andensis* (Hambleton) (Caballero et al. 2019) and *R.colombiensis* (Hambleton) (Smith et al. 2007). In recent years, we have discovered several cryptic species of *Ripersiella* collected from nests of *Acropyga* ants in the Neotropical region. Each of the cryptic species is similar in appearance to *R.andensis* or *R.colombiensis* and they each key out as one of these two species using the best available identification tools (i.e., Williams and Granara de Willink 1992; Kozár and Konczné Benedicty 2007; Szita et al. 2020). It is intriguing to discover a complex of similar-looking *Ripersiella* species all associated with *Acropyga* ants. If this complex forms a monophyletic clade, such a result would offer the first evidence that a species radiation of rhizoecid trophobionts has taken place, independent from the radiation of Xenococcidae. Conversely, if they are non-monophyletic, this may suggest that living with *Acropyga* results in phenotypic convergence among trophobiotic root mealybugs that are somewhat distant relatives. Phylogenetic analysis and further testing of these competing hypotheses is underway.

In this article, we describe five new species of *Ripersiella* from Peru and the Dominican Republic. In combination with *R.andensis* and *R.colombiensis*, these seven species form a complex that we refer to informally here as the *andensis*-complex. Furthermore, we provide a key to the Neotropical species of *Ripersiella* which lack bitubular cerores to aid in the identification of species in the *andensis*-complex. A thorough treatment of the *Ripersiella* species from the Neotropical region was recently provided by Szita et al. (2020) and is therefore not repeated here. However, we do include descriptions and re-illustrations of *R.andensis* and *R.colombiensis* along with comments on their affiliation with *Acropyga*.

Many reports on the trophobiosis between *Acropyga* ants and scale lineages falling outside of Xenococcidae require verification to confirm that the ant and scale species were in fact directly associated (see Schneider and LaPolla 2020; Schneider et al. 2022). When excavating *Acropyga* nests we sometimes find free-living hypogeic scale insects neighboring the colony, but the ants show no interest in these individuals and workers do not collect honeydew from them. Some published reports, which included explicit documentation verifying direct species-to-species association, are unequivocal (Smith et al. 2007; LaPolla et al. 2008; Tanaka 2016; Schneider and LaPolla 2020). Here, we detail the evidence and methods that were used to confirm direct association between scale insect species and *Acropyga* ants or state when such evidence is lacking.

## ﻿Methods

Specimens were preserved in 95–100% ethanol and stored at −80 °C prior to preparation. Examined specimens were prepared either by slide mounting directly or by first extracting DNA prior to mounting their cuticle. Extractions were performed using the QIAamp DNA Mini Kit (Qiagen, Valencia, California) following the standard protocol; specimen cuticles were removed from the extraction buffer after the initial lysing step and subsequently mounted. DNA-extracted specimens were assigned a unique six-digit alphanumeric identifier beginning with “S” followed by four numbers used to identify the collection event and ending with a sequentially assigned letter to identify the individual specimen (e.g., S0439A). Their DNA extractions, preserved at −80 °C, are housed at the USDA ARS Beltsville Agricultural Research Center, Beltsville, Maryland, USA. Individuals that were slide-mounted directly are identified with a sequentially assigned letter appended to the end of the collector number (e.g., JSL090804-05A). All specimens were slide-mounted following the protocol described in Normark et al. (2019).

The terminology used in this paper follows Hambleton (1946), Kozár and Konczné Benedicty (2007), and Williams and Granara de Willink (1992). Measurements were made on a Zeiss Axio Imager.M2 (Carl Zeiss Microscopy, LLC, White Plains, NY, USA) microscope with the aid of an AxioCam and AxioVision software. Slide-mounted specimens were examined under phase contrast and differential interference contrast. Cryo-SEM was carried out at the US Department of Agriculture, Electron and Confocal Microscopy Unit (Beltsville, MD, USA), using a Hitachi SU-7000 + Quorum PP3010 Cryo Prep System + Oxford X-Max EDS field emission scanning electron microscope (Hitachi High Technologies America, Pleasanton, CA, USA). Images were captured and processed using the techniques described in Bolton et al. (2014).

Identification of associated *Acropyga* ants was performed using the key to species provided by LaPolla (2004).

Type depositories are abbreviated as follows:

**MNHNSD**Museo Nacional de Historia Natural Prof. Eugenio de Jesús Marcano, Santo Domingo, Dominican Republic;

**UNAB**Museo Entomológico Facultad de Agronomía, Universidad Nacional de Colombia, Bogotá, Colombia;

**UKNMH**Natural History Museum, London, United Kingdom;

**UNMSM**Museo de Historia Natural, Universidad Nacional Mayor de San Marcos, Lima, Peru;

**USNM**Smithsonian National Museum of Natural History, Coccomorpha collection at USDA Agricultural Research Service, Beltsville, Maryland, USA.

For our collections from Peru, we confirmed direct trophobiotic association between root mealybugs and *Acropyga* ants through careful observation of interacting partners using a nest-box, following the protocol described by Schneider et al. (2022). For collections from the Dominican Republic, we confirmed direct association through observations in the field; their association is further evidenced through repeated collection of the same species pairs from nests at multiple sites. One new species described here was collected from Peru by T.R. Schultz. Exercising an abundance of caution, we consider this association as likely but needing confirmation, since specimens were collected from a single nest and the field notes lacked details on how direct association was confirmed.

## ﻿Taxonomy

### ﻿Genus Ripersiella

Taxon classificationAnimaliaHemipteraRhizoecidae

Tinsley, 1899

CF9C2425-E6C0-5CFE-AD05-2D6A659D371F

Ripersiella Tinsley in Cockerell, 1899: 278. Type species: Ripersiarumicis Maskell, 1892.
Rhizoecus (Pararhizoecus) Goux, 1941: 197. Type species: Rhizoecuspetiti Goux, 1941.
Pararhizoecus Goux, 1941; Goux 1943: 41.

#### Remark.

The new species described below are placed in *Ripersiella* based on the following diagnosis, which is a condensed version of the comprehensive descriptions provided by Kozár and Konczné Benedicty (2007) and Szita et al. (2020). For further details on the genus and a broader treatment of species, refer to these references.

#### Diagnosis.

Tritubular cerores (also referred to as tritubular pores or ducts) absent; bitubular cerores (bitubular pores/ducts) typically present, absent in some species; anal ring with or without elongate cells, lacking protuberances, and situated dorsally; body setae all flagellate, anal lobes usually poorly developed, bearing a set of 3 distinct long setae or with several short setae; trilocular pores present but never arranged in tight clusters on the venter; body oval to spherical and membranous; antennae geniculate with 5 or 6 segments.

#### Comments.

In Kozár and Konczné Benedicty (2007) and Szita et al. (2020), species that lack bitubular cerores but are otherwise morphologically similar to the generic type species, *R.rumicis* (Maskell), have been tentatively placed in *Ripersiella*. We maintain the established precedent here. However, it is important to note that Choi and Lee (2022), in their phylogenetic analysis of mealybug clades, failed to recover a monophyletic *Ripersiella*, and our own preliminary phylogenomic analyses (unpublished data) show similar results. It is therefore likely that some or all of the new species described here will eventually require a change of combination corresponding with a revision of Rhizoecidae that is informed through both their morphology and molecular phylogenetic analysis.

### ﻿ Ripersiella andensis

Taxon classificationAnimaliaHemipteraRhizoecidae

(Hambleton)

4CCFB915-338B-51CE-AE5F-AD48581BC660

Fig. 1

Neorhizoecus andensis Hambleton, 1946: 41.
Rhizoecus andensis (Hambleton); Hambleton 1977: 369.
?Ripersiellaandensis (Hambleton); Kozár and Konczné Benedicty 2003: 235.

#### Material examined.

***Lectotype*.** Colombia • 1 adult ♀; Bogota; 22.ii.1935; L.M. Murillo; on roots of *Coffeaarabica* L.; USNM. ***Paralectotypes*.** Colombia • 2 adult ♀♀; same slide as lectotype; USNM • 3 adult ♀♀; same data as lectotype; USNM. ***Other material*.** Colombia • 4 adult ♀♀; locality (?); 1955; D. Rios Castana; on coffee; USNM • 3 adult ♀♀; locality (?); iv.1956; S.G. Flanders; on coffee; USNM • 22 adult ♀♀; Chinchina Cald.; 18.xii.1975; R. Cardenas; USNM.

**Figure 1.**
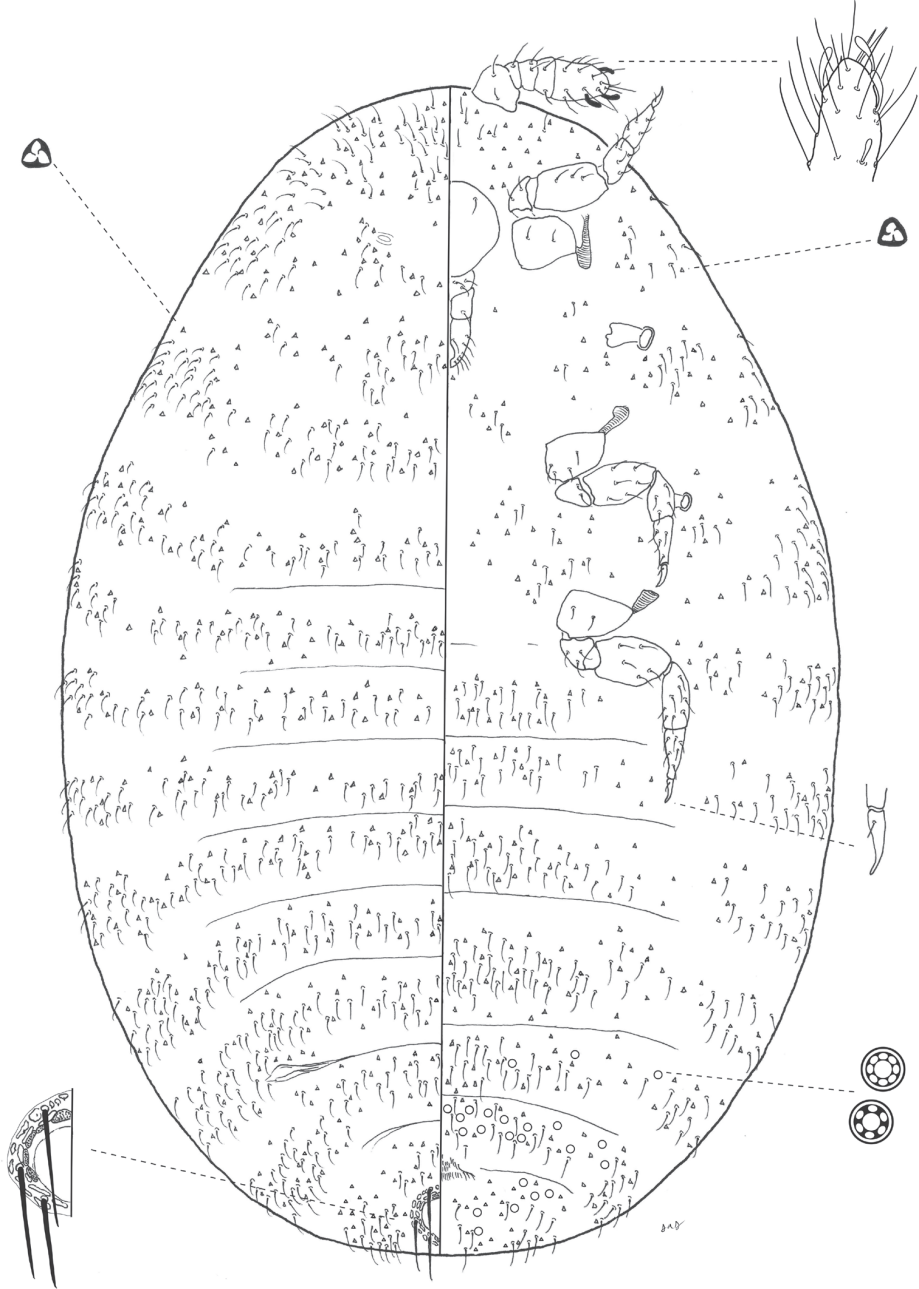
*Ripersiellaandensis* (Hambleton). Adult female, full body view, re-illustrated by SAS from Williams and Granara de Willink (1992) and Kozár and Konczné Benedicty (2007).

#### Description.

Adult female (*N* = 6). Appearance in life not recorded, extent of wax production unknown.

**General.** Mounted on microscope slide, body broadly oval and membranous, 0.78–1.06 (0.98) mm long, widest near abdominal segments II–III, 0.41–0.58 (0.52) mm wide. Abdomen smoothly tapering toward apex; abdominal segment VIII 170–213 (180) μm wide at base. Anal lobes poorly developed with several flagellate setae on venter and dorsum, ranging from 15–33 μm long. Body setae flagellate, 10–22 μm on head, 9–15 μm on thorax, 11–17 μm on abdominal segments. Trilocular pores abundant and distributed among body setae; bitubular cerores absent; oral collar tubular ducts absent. Microtrichia present on abdominal segments. Eyes absent.

***Venter*.** Cephalic plate absent. Labium with 3 segments; 70 μm long and 46 μm at widest point. Antennae geniculate, 5-segmented, closely situated near midline on ventral submargin of head; overall length 126–134 (132) μm; length of segment I: 27–38 (34) μm; segment II: 14–17 (16) μm; segment III: 14–16 (15) μm; segment IV: 13–18 (14) μm; segment V: 52–53 (53) μm; apical antennal segment with 1 spine-like and 4 falcate stout sensory setae; few flagellate setae on each antennal segment, 14–35 μm long; sensorium present on second antennal segment. Legs well developed; overall length of hind leg 235–241 (238) μm; length of hind coxa 28–34 (34) μm; hind trochanter + femur 91–95 (91) μm; hind tibia + tarsus 88–91 (91) μm; hind claw 22–25 (22) μm; each claw with short setose digitule 2–3 μm long; flagellate setae present on each segment, around 19 μm long. Circulus absent. Multilocular disc pores in irregular rows or groups on segments VI–VIII, pores with 6–8 loculi in the outer ring.

***Dorsum*.** Anal ring on dorsal surface near posterior margin, 48–52 μm in diameter; with oval cells, some cells bearing spicules; with three pairs of setae 33–39 μm long. Posterior pair of dorsal ostioles present, diameter of orifice measured along longitudinal axis approximately 45 μm; anterior pair present but much smaller than posterior pair and poorly developed, diameter of orifice approximately 10 μm. Multilocular disc pores absent.

#### Diagnosis.

The following newly described species are similar in appearance to *R.andensis*: *R.campensis*, *R.montanae*, and *R.pediandensis*. Consult the respective diagnosis sections under each species below for a discussion on how to distinguish them from *R.andensis*. Another new species, *R.telalia*, can easily be distinguished by the presence of well-developed anterior ostioles, which are present in *R.andensis* but are strongly reduced and may appear to be absent.

#### Comments.

*Ripersiellaandensis* may associate with *Acropygaexsanguis* and *A.smithii* (Caballero et al. 2019); however, confirmation of their direct association is lacking, and Schneider and LaPolla (2020) considered their reported trophobiosis to be speculative. Given that there is a complex of species resembling *R.andensis* living in association with *Acropyga*, it seems likely that they do associate, and this simply requires confirmation. It would be useful to clearly establish whether there are both free-living and ant-associated populations as well.

### ﻿ Ripersiella campensis

Taxon classificationAnimaliaHemipteraRhizoecidae

Schneider & LaPolla sp. nov.

D18BCC79-3B79-5EA2-8671-2BAA67E2750C

https://zoobank.org/5FACC224-2788-4034-A03B-83D0C95F321D

Figures 2 , 3

#### Material examined.

***Holotype*.** Dominican Republic • 1 adult ♀; Loma Novillero (Fonestal Reserva) near Villa Altagracia; 18.7032, -70.1931, elev. 187 m; 4.viii.2009; JS LaPolla, SA Schneider leg.; associated with *Acropygadubitata*, nest in 2° forest at base of tree root; USNM (nest DR8: prep JSL090804-05A). ***Paratypes*.** Dominican Republic • 1 adult ♀; same data as holotype; USNM (nest DR8: prep S0439A) • 1 adult ♀; same data as holotype; UNAB (nest DR8: prep JSL090804-05B) • 1 adult ♀; Rancho Capote near Hato Mayor, 18.7971, -69.4194, elev. 112 m; 3.viii.2009; JS LaPolla, SA Schneider leg.; associated with *Acropygadubitata*, nest under large tree root in riparian forest near Fun-Fun Cave; USNM (nest DR6: prep JSL090803-05A) • 1 adult ♀; same data as previous; UNAB (nest DR6: prep JSL090803-05B) • 1 adult ♀; San Francisco Mountains, Loma Quita Espuela Reserve, 19.3386, -70.1482, elev. 290 m; 30.vii.2009; JS LaPolla, SA Schneider leg.; associated with *Acropygadubitata* in mixed forest/cacao plantation, host *Theobroma* sp.; MNHNSD (nest DR3: prep JSL090730-08A) • 4 adult ♀♀; same data as previous; USNM (nest DR3: preps S0436A; JSL090730-05B,C; JSL090730-08D) • 1 adult ♀; San Francisco Mountains, Loma Quita Espuela Reserve, 19.3386, -70.1482, elev. 290 m; 31.vii.2009; JS LaPolla, SA Schneider leg.; associated with *Acropygadubitata* in mixed forest/cacao plantation, host *Theobroma* sp.; UKMNH (nest DR4: prep JSL090731-01A) • 1 adult ♀; same data as previous; MNHNSD (nest DR4: prep JSL090731-01B) • 1 adult ♀; same data as previous; UKNMH (nest DR4: prep JSL090731-01C) • 3 adult ♀♀; same data as previous; USNM (nest DR4: preps JSL090731-02D,E,F) • 1 adult ♀; same data as previous; USNM (nest DR5: prep S0437A).

**Figure 2.**
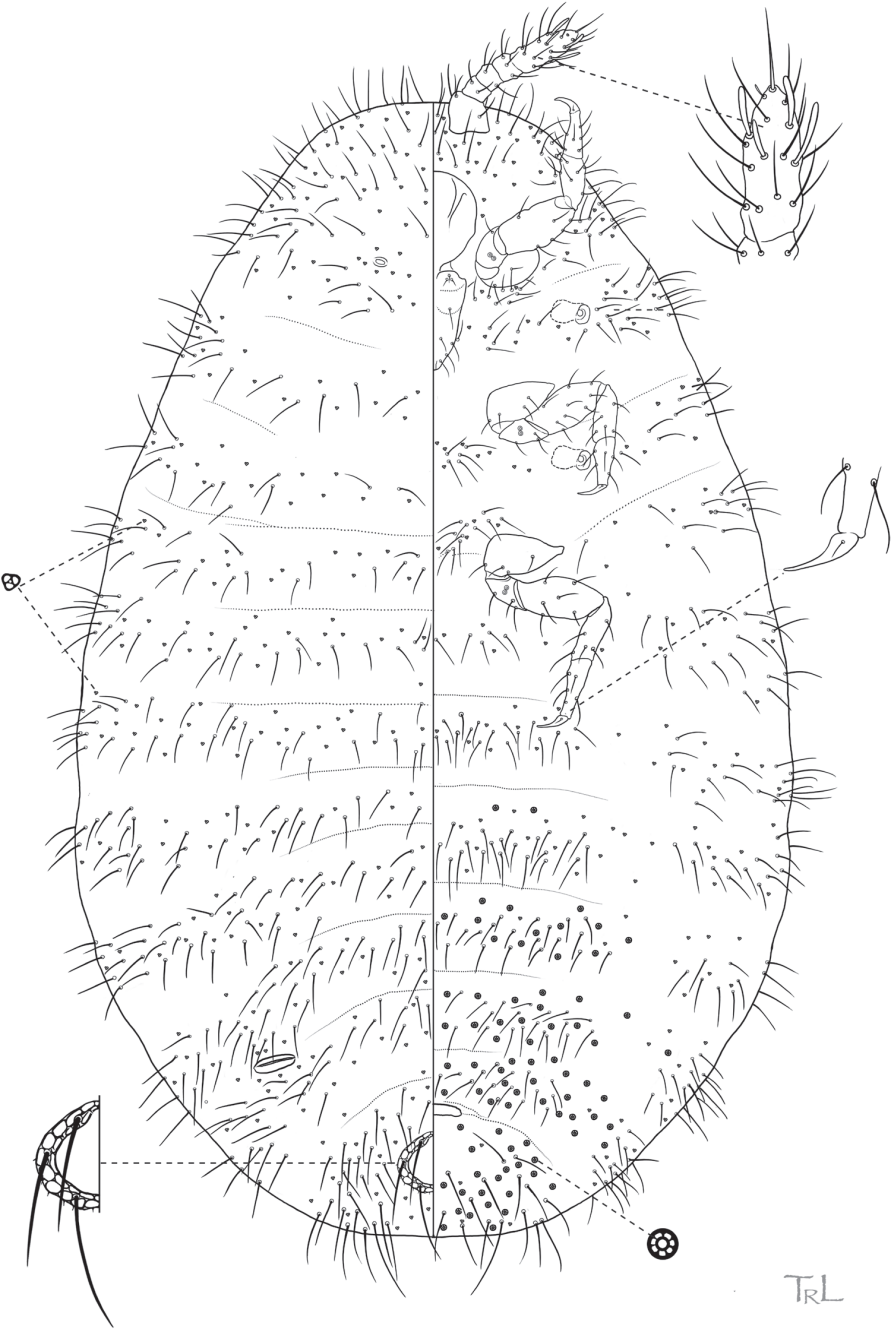
*Ripersiellacampensis* Schneider & LaPolla sp. nov. Adult female, full body view, illustrated from holotype by T. Litwak (USDA ARS SEL), with edits by SAS.

#### Description.

**Figure 3.**
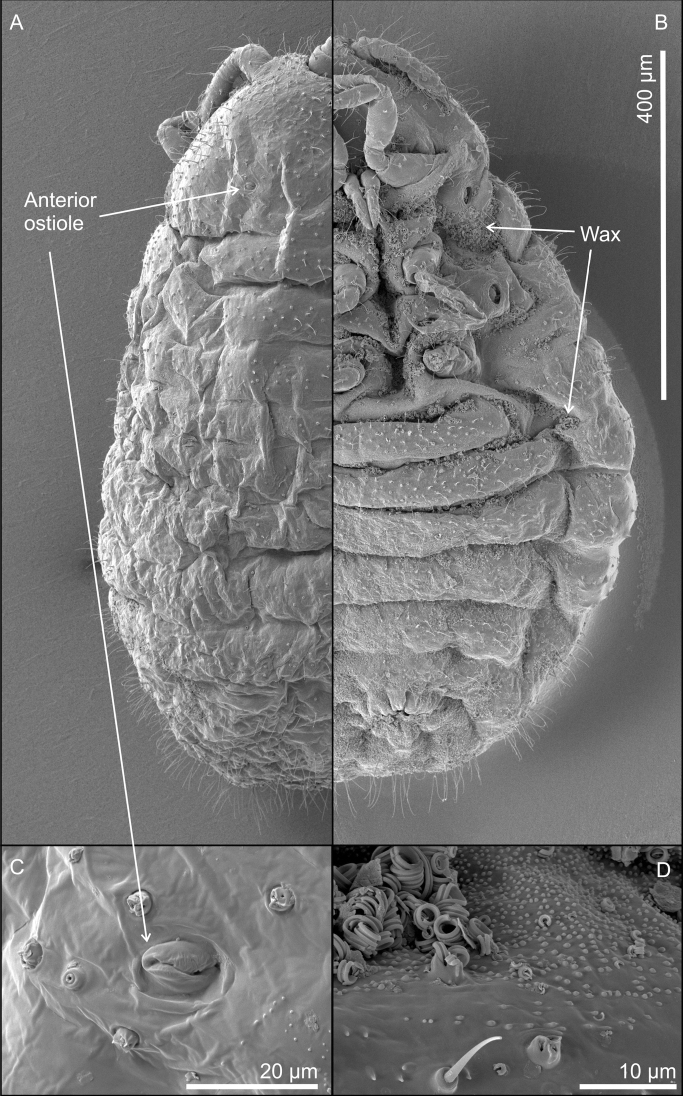
*Ripersiellacampensis* Schneider & LaPolla sp. nov. Scanning electron micrographs (SEM) by J. Mowery (USDA ARS SEL) **A** dorsal view **B** ventral ½ view of adult female **C** magnified view of miniscule anterior dorsal ostiole **D** magnified view of curled wax deposits, trilocular pores, spine-like microtrichia, and abundant domed dermal micro-bumps in intersegmental region.

Adult female (*N* = 17). In life, body bright white to cream colored and free of obvious waxy secretions, small deposits of wax from trilocular pores visible under SEM (Fig. 3), tending to gather in intersegmental regions of the body and appendages.

**General.** Mounted on microscope slide, body broadly oval and membranous, 0.86–1.03 (0.93) mm long, widest near abdominal segments III–V, 0.50–0.70 (0.60) mm wide. Abdomen slightly constricted between segments VII and VIII or smoothly tapering; abdominal segment VIII 186–251 (210) μm wide at base. Anal lobes poorly developed with several flagellate setae on venter and dorsum, ranging from 23–75 μm long, with 1 or 2 longer setae situated near dorsal margin. Body setae flagellate, 14–40 μm on head, 13–33 μm on thorax, 14–37 μm on abdominal segments. Trilocular pores abundant and distributed among body setae; bitubular cerores absent; oral collar tubular ducts absent. Microtrichia present on abdominal segments; rounded dermal micro-bumps abundant in intersegmental areas and surroundings of appendages. Eyes absent.

***Venter*.** Cephalic plate absent. Labium with 3 segments; 71 μm long and 43 μm at widest point. Antennae geniculate, 5-segmented, closely situated near midline on ventral submargin of head; overall length 140–156 (146) μm; length of segment I: 33–43 (34) μm; segment II: 17–21 (17) μm; segment III: 14–19 (16) μm; segment IV: 14–19 (18) μm; segment V: 53–60 (58) μm; apical antennal segment with 1 spine-like seta, 4 falcate stout sensory setae, and what appears to be 1 minute sensory seta at terminal apex; few flagellate setae on each antennal segment, 25–35 μm long; sensorium present on second antennal segment. Legs well developed; overall length of hind leg 266–292 (280) μm; length of hind coxa 34–49 (34) μm; hind trochanter + femur 101–115 (103) μm; hind tibia + tarsus 103–108 (105) μm; hind claw 23–27 (26) μm; each claw with short setose digitule 2–3 μm long; flagellate setae present on each segment, around 27 μm long. Circulus absent. Multilocular disc pores in irregular rows or groups on segments III–VIII, sometimes missing from segments III or IV but always present at least as far anterior as V, pores with 7 loculi in the outer ring.

***Dorsum*.** Anal ring on dorsal surface separated from posterior body margin by approximately 1× diameter of ring, 49–59 μm in diameter; with oval cells, some cells bearing spicules; with 3 pairs of setae 40–54 μm long. Posterior pair of dorsal ostioles present, diameter of orifice measured along longitudinal axis approximately 45 μm; anterior pair present but much smaller than posterior pair and poorly developed, diameter of orifice approximately 10 μm. Multilocular disc pores absent.

#### Informal synonyms.

Specimens of *R.campensis* have been previously referred to in the literature as “*Rhizoecus* new sp.” (Schneider and LaPolla 2011). At the time, collections from the Dominican Republic were thought to comprise a single species associated with *A.dubitata* and the generic assignment was uncertain.

#### Etymology.

The species epithet is an adjective formed from the Latin noun *campus* referring to “a level place or surface” with the suffix -*ensis* denoting “of or from a place” alluding to the type series being collected only from lowland areas of Hispaniola.

#### Diagnosis.

*Ripersiellacampensis* sp. nov. is similar in appearance to *R.montanae* sp. nov., described below. Morphological differences between the two species are subtle, but they can be distinguished based on the following suite of characteristics. In *R.campensis*, multilocular disc pores are present on abdominal segments V–VIII and usually present on segments III–IV as well, body setae are comparatively longer and sparsely distributed, antennal segments II–IV are subequal in length (average lengths in μm: 19, 16, 17), and segment V is approximately 57 μm long. In *R.montanae*, multilocular disc pores are restricted to abdominal segments VI–VIII, body setae are comparatively shorter and densely distributed, antennal segments II–IV differ in length (average lengths in μm: 13, 24, 20), and segment V is approximately 40 μm long.

*Ripersiellacampensis* is also similar in appearance to *R.andensis*. The two species can be distinguished as follows (character states for *R.andensis* are given in parentheses): having multilocular disc pores on any of segments III–V (absent on these segments); having anal lobe setae as long as 75 μm (as long as 35 μm); setae on the head, thorax, and abdomen are exceeding 30 μm (not exceeding 25 μm); hind legs are approximately 280 μm long (240 μm); and antennae are approximately 146 μm long (128 μm).

#### Comments.

*Ripersiellacampensis* was discovered from five nests of *Acropygadubitata* (Wheeler & Mann) (nests DR3–6,8). The nests were located in lowland (between 112–290 m) forested areas, including a mixed forest/cacao plantation, riparian forest, and secondary growth forest near agricultural fields. We verified direct species-to-species association (trophobiosis) between the scale insects and ants through observation of attendance by worker ants and by the fact that all colonies contained numerous individuals of the same root mealybug species within their nest chambers and no additional species of scale insects were present. In the Dominican Republic, *R.montanae* also associates with *A.dubitata* but potentially only in areas of high elevation (>1000 m) in the mountains near the shared border with Haiti.

### ﻿ Ripersiella colombiensis

Taxon classificationAnimaliaHemipteraRhizoecidae

(Hambleton)

62993AF4-2C17-5FD4-A268-0988B45DC7A7

Figure 4

Neorhizoecus colombiensis Hambleton, 1946: 43.
Rhizoecus colombiensis (Hambleton); Hambleton 1977: 372.
Ripersiella colombiensis (Hambleton); Kozár and Konczné Benedicty 2003: 236.

#### Material examined.

***Holotype*.** Colombia • 1 adult ♀; La Esperanza; ii.1936; R Roba coll.; USNM. ***Other material*.** United States • 1 adult ♀; Arizona, Cochise Co., Chiricahua Mtns, SW Res. Sta., 5 miles W. Portal; 31.8833, -109.2063, 1646 m; 5–15.viii.2001; JS LaPolla; with *Acropygaepedana*; USNM • 1 adult ♀; Arizona, Cochise Co., near Portal; 31.8838, -109.2229, 1645 m; 31.vii.2005; CR Smith; collected from colony of *Acropygaepedana*; USNM.

**Figure 4.**
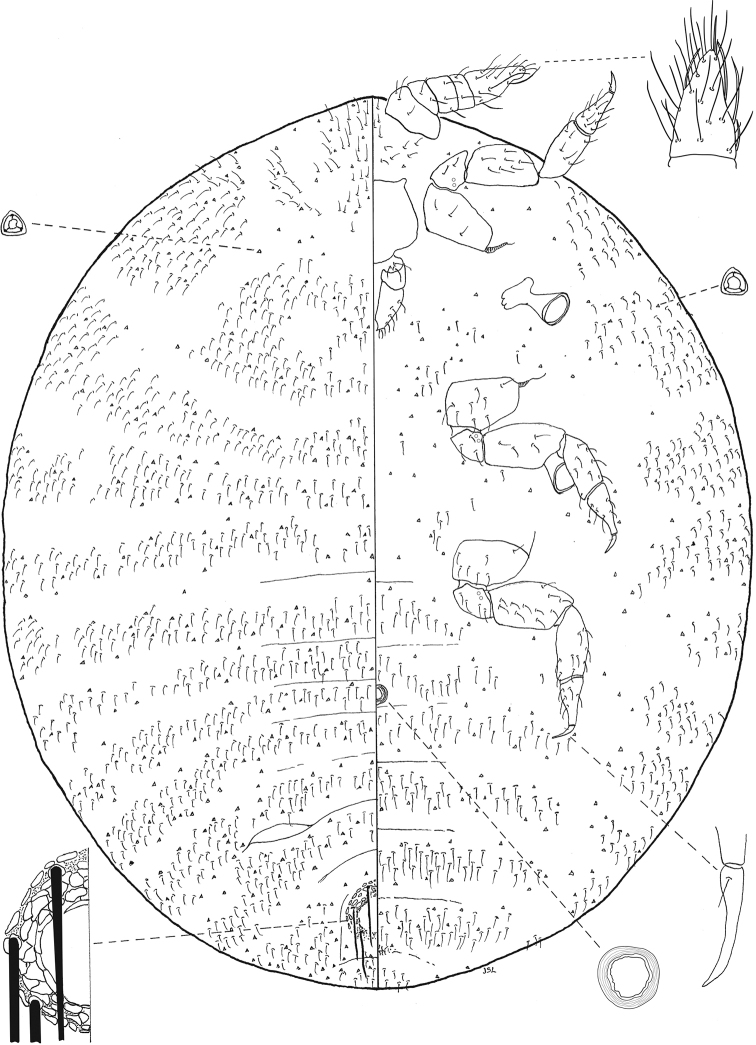
*Ripersiellacolombiensis* (Hambleton). Adult female, full body view, re-illustrated by JSL from Williams and Granara de Willink (1992) and Kozár and Konczné Benedicty (2007), with edits by SAS.

#### Description.

Adult female, based on holotype. Appearance in life not reported; extent of wax production unknown.

**General.** Mounted on microscope slide, body broadly oval and membranous, 1.09 mm long, widest at metathorax and abdominal segments I–II, 0.91 mm wide. Abdomen rounded and gently tapering toward posterior apex; abdominal segment VIII approximately 270 μm wide at base. Anal lobes poorly developed with several flagellate setae on dorsum and venter, 18–28 μm long; lacking differentiated group of 3 long anal lobe setae. Body setae flagellate, 17–25 μm on head, 15–20 μm on thorax, 16–21 μm on abdominal segments. Trilocular pores scarcely distributed among body setae; multilocular disc pores absent; bitubular cerores absent; oral collar tubular ducts absent. Microtrichia present on abdominal segments and thorax. Eyes absent.

***Venter*.** Cephalic plate absent. Labium with 3 segments; 88–110 μm long and 69 μm at widest point. Antennae geniculate, 5-segmented, closely situated near midline on ventral submargin of head; overall length 182 μm; length of segment I: 36 μm; segment II: 26 μm; segment III: 28 μm; segment IV: 22 μm; segment V: 70 μm; apical segment with 1 spine-like and 4 falcate sensory setate; flagellate setae on each antennal segment 18–40 μm long. Legs well developed; overall length of hind leg 394 μm; length of hind coxa 48 μm; length of hind trochanter + femur 158 μm; length of hind tibia + tarsus 152 μm; length of hind claw 36 μm; each claw with setose digitule 4 μm long; flagellate or stout setae present on each segment 18–28 μm long. Single conical circulus present between abdominal segments III and IV, 28 μm wide; inner margin of orifice slightly crenulated.

***Dorsum*.** Anal ring situated on dorsal surface separated from posterior body margin by approximately 1× diameter of ring, 80 μm in diameter; with oval cells lacking spicules; bearing three pairs of setae about 63 μm long. Posterior pair of ostioles present, diameter of orifice measured along longitudinal axis approximately 68 μm; anterior pair of ostioles apparently absent.

#### Diagnosis.

*Ripersiellacolombiensis* is most similar in appearance to the newly described species *R.illicians*. Consult the diagnosis of *R.illicians* for a discussion on how to distinguish them.

#### Comments.

*Ripersiellacolombiensis* is confirmed to associate with *Acropygaepedana* and is one of few species of Rhizoecidae with a published record of *Acropyga* queens carrying gravid female trophobionts on their nuptial flight (Smith et al. 2007; Schneider and LaPolla 2020). Specimens from Colombia and Arizona appear likely to be conspecific, although those from Arizona have noticeably longer and thinner legs relative to their body size.

### ﻿ Ripersiella illicians

Taxon classificationAnimaliaHemipteraRhizoecidae

Schneider & LaPolla sp. nov.

C40AB79F-BC44-59E2-ADE9-335BDF63398D

https://zoobank.org/7A49B97A-43A7-48D4-B8DF-8D9408E9A882

Figures 5 , 6

#### Material examined.

***Holotype*.** Peru • 1 adult ♀; Madre de Dios, Manu National Park, Cocha Cashu Biological Station, near trail marker 27:1150; -11.8833, -71.4000; 10.vi.2019; JS LaPolla, SA Schneider leg.; upland forest, from large nest of *Acropygagoeldii* (group) at base of tree; USNM (nest PER25-01: prep S0426E). ***Paratypes*.** Peru • 3 adult ♀♀; same data as holotype; USNM (nest PER25-01: preps S0426B,D,F) • 1 adult ♀; same data as holotype; UNMSM (nest PER25-01: prep S0426C) • 1 adult ♀; same data as holotype; UNAB (nest PER25-01: prep S0426G) • 1 adult ♀; same data as holotype; UKMNH (nest PER25-01: prep S0426A).

**Figure 5.**
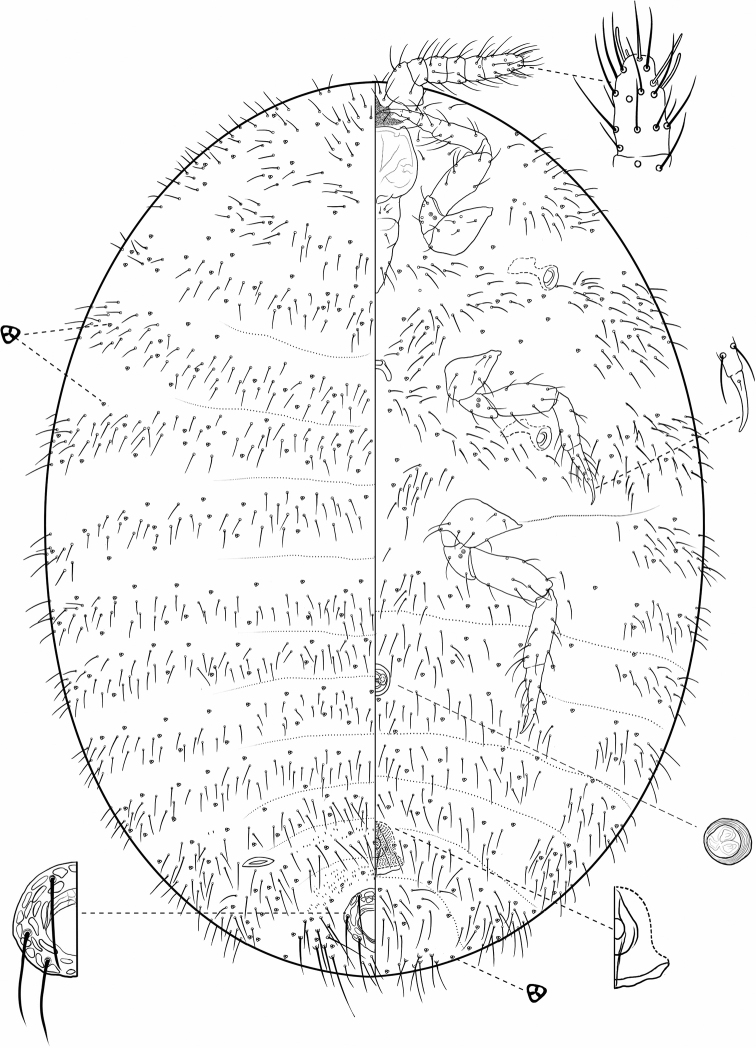
*Ripersiellaillicians* Schneider & LaPolla sp. nov. Adult female, full body view, illustrated from holotype by T. Litwak (USDA ARS SEL), with edits by SAS.

#### Description.

Adult female (*N* = 7). In life, body bright white to cream colored and visibly coated in powdery white wax.

**Figure 6.**
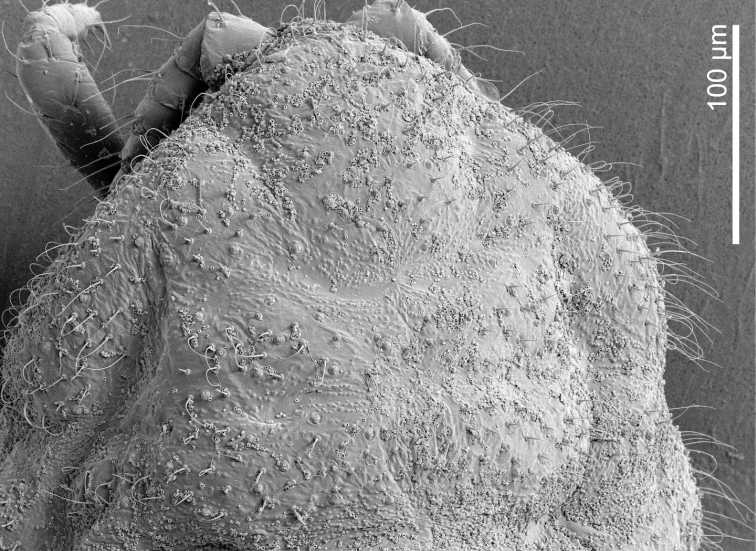
*Ripersiellaillicians* Schneider & LaPolla sp. nov. Scanning electron micrograph (SEM) by J. Mowery (USDA ARS SEL). Dorsal anterior view of adult female head and thorax, showing the absence of anterior ostioles and the presence of curled wax deposits and domed dermal micro-bumps in intersegmental areas.

**General.** Mounted on microscope slide, body broadly oval and membranous, 0.70–0.83 (0.78) mm long, widest at metathorax and abdominal segments I–II, 0.46–0.61 (0.55) mm wide. Abdomen rounded and gently tapering toward posterior apex; abdominal segment VIII 148–196 (196) μm wide at base. Anal lobes poorly developed with several flagellate setae on dorsum, 57–60 μm long. Body setae flagellate, 15–27 μm on head, 17–33 μm on thorax, 15–40 μm on abdominal segments. Trilocular pores abundant and distributed among body setae; multilocular disc pores absent; bitubular cerores absent; oral collar tubular ducts absent. Microtrichia apparently present only on dorsum of segments VI–VIII; rounded dermal micro-bumps present in intersegmental areas and surroundings of appendages. Internal genital organ sclerotized in some specimens of type series. Eyes absent.

***Venter*.** Cephalic plate present. Labium with 3 segments; 82 μm long and 41 μm at widest point. Antennae geniculate, 6-segmented, closely situated near midline on ventral submargin of head; overall length 130–151 (151) μm; length of segment I: 30–37 (37) μm; segment II: 14–18 (18) μm; segment III: 17–20 (20) μm; segment IV: 14–19 (19) μm; segment V: 13–17 (17) μm; segment VI: 39–40 (40) μm; apical segment with 4 falcate stout sensory setate; a few flagellate setae present on each antennal segment, 19–30 μm long. Legs well developed; overall length of hind leg 274–290 (288) μm; length of hind coxa 43–44 (44) μm; length of hind trochanter + femur 102–108 (106) μm; length of hind tibia + tarsus 102–110 (110) μm; length of hind claw 26–31 (28) μm; each claw with stout setose digitule 1–2 μm long; flagellate setae present on each segment approximately 23 μm long. Single conical circulus present between abdominal segments III and IV, 22 μm in diameter; inner margin of orifice crenulated or with rugose projections.

***Dorsum*.** Anal ring situated on dorsal surface separated from posterior body margin by approximately one-half diameter of ring, 59–61 μm in diameter; with oval cells lacking spicules; bearing 3 pairs of setae about 50 μm long. Posterior pair of ostioles present, diameter of orifice measured along longitudinal axis approximately 39 μm; anterior pair of ostioles absent.

#### Informal synonyms.

Specimens of *R.illicians* have been previously referred to in the literature as “*Ripersiella* undescribed (i)” (Schneider et al. 2022).

#### Etymology.

The species epithet is an adjective formed from *illicium*, meaning attraction or enticement, and its use alludes to the apparent tendency of *Acropyga* colonies to gain rhizoecid partners through horizontal acquisitions. Such colonies have been figuratively attracted away from their primary associates in Xenococcidae.

#### Diagnosis.

*Ripersiellaillicians* sp. nov. is similar to *R.colombiensis* as both species have a subcircular body shape, bearing one circulus, and both are lacking multilocular disc pores and an anterior pair of dorsal ostioles. However, *R.illicians* can be distinguished from *R.colombiensis* as follows (character states for *R.colombiensis* are given in parentheses): having 6-segmented antennae (5-segmented antennae); having comparatively long body setae, ranging from 15–40 μm (comparatively short, ranging from 15–25 μm); and having anal lobe setae that are distinctly longer than body setae, 57–60 μm (similar in length to body setae, 18–28 μm).

#### Comments.

*Ripersiellaillicians* was discovered from a large nest of *Acropygagoeldii* (group). Root mealybugs were abundant in the nest, and wax could be seen on their body using a hand lens. Their direct association was confirmed through observation of the colony using a nest-box, as described by Schneider et al. (2022). After specimens were collected into a nest-box, worker ants gathered trophobionts into a protective cluster and were actively engaged in attending to them.

### ﻿ Ripersiella montanae

Taxon classificationAnimaliaHemipteraRhizoecidae

Schneider & LaPolla sp. nov.

480B988A-E17E-5E0D-A434-7BE270887651

https://zoobank.org/82F8E9C9-49A4-4436-A8C6-9817CBE46D76

Figures 7 , 8

#### Material examined.

***Holotype*.** Dominican Republic • 1 adult ♀; W. of Hondo Valley, 13 m off road; 18.7229, -71.7061, elev. 1032 m; 24.vii.2009; JS LaPolla, SA Schneider leg.; associated with *Acropygadubitata*, nest under a stone in coffee plantation next to road, host *Coffea* sp.; USNM (nest DR2: prep JSL090724-13A). ***Paratypes***. Dominican Republic • 5 adult ♀♀; same data as holotype; USNM (nest DR1: preps S0434A; S0435A; JSL090724-08A,B; JSL090724-10F) • 1 adult ♀; same data as holotype; MNHNSD (nest DR1: prep JSL090724-05E) • 1 adult ♀; same data as holotype; UNAB (nest DR1: prep JSL090724-08C) • 1 adult ♀; same data as holotype; UKMNH (nest DR1: prep JSL090724-08D).

**Figure 7.**
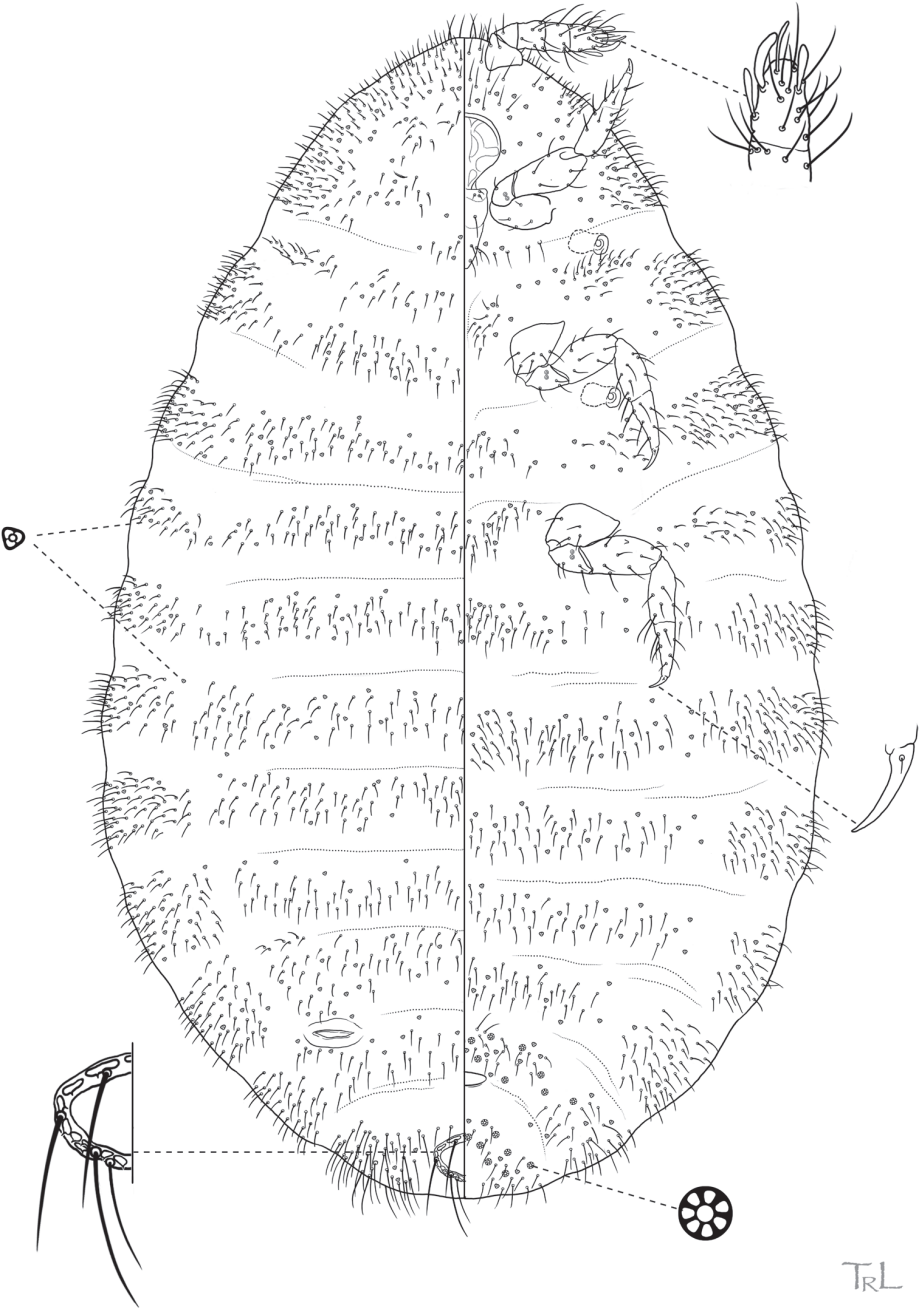
*Ripersiellamontanae* Schneider & LaPolla sp. nov. Adult female, full body view, illustrated from holotype by T. Litwak (USDA ARS SEL), with edits by SAS.

#### Description.

**Figure 8.**
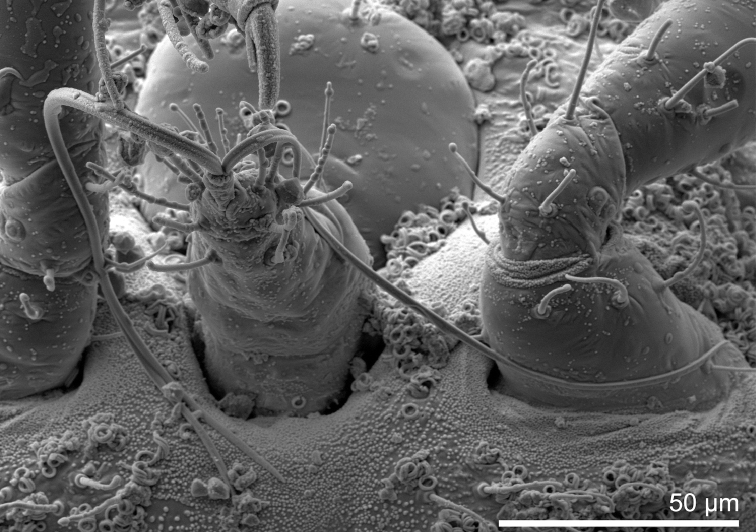
*Ripersiellamontanae* Schneider & LaPolla sp. nov. Scanning electron micrograph (SEM) by J. Mowery (USDA ARS SEL). Ventral view of adult female labium and prothoracic legs, showing curled wax deposits and an abundance of domed dermal micro-bumps surrounding the attachment points of appendages.

Adult female (*N* = 9). In life, body bright white to cream colored and free of obvious waxy secretions, small deposits of wax from trilocular pores visible under SEM (Fig. 8), tending to gather in intersegmental regions of the body and appendages.

**General.** Mounted on microscope slide, body broadly oval and membranous, 0.91–1.03 (0.99) mm long, widest near abdominal segments III–V, 0.51–0.68 (0.62) mm wide. Abdomen slightly constricted between segments VII and VIII or smoothly tapering; abdominal segment VIII 199–250 (199) μm wide at base. Anal lobes poorly developed with several flagellate setae on venter and dorsum, ranging from 16–59 μm long, with longest setae situated near dorsal margin; lacking differentiated set of 3 longer stout setae common to the group. Body setae short and flagellate, 10–22 μm on head, 12–20 μm on thorax, 14–24 μm on abdominal segments. Trilocular pores abundant and distributed among body setae; bitubular cerores absent; oral collar tubular ducts absent. Microtrichia present on abdominal segments; rounded dermal micro-bumps abundant in intersegmental areas and surroundings of appendages (Fig. 8). Eyes absent.

***Venter*.** Cephalic plate absent. Labium with 3 segments; 70 μm long and 42 μm at widest point. Antennae geniculate, 5-segmented, closely situated near midline on ventral submargin of head; overall length 126–133 (129) μm; length of segment I: 30–34 (34) μm; segment II: 12–19 (13) μm; segment III: 20–26 (24) μm; segment IV: 17–21 (18) μm; segment V: 36–42 (40) μm; apical antennal segment with 1 spine-like and 4 falcate stout sensory setae; a few flagellate setae on each antennal segment, 20–30 μm long; sensorium present on second antennal segment. Legs well developed; overall length of hind leg 253–277 (275) μm; length of hind coxa 32–48 (48) μm; hind trochanter + femur 86–101 (95) μm; hind tibia + tarsus 101–111 (111) μm; hind claw 21–26 (21) μm; each claw with short setose digitule 2–3 μm long; flagellate or stout setae present on each segment, about 25 μm long. Circulus absent. Multilocular disc pores in irregular rows or groups on segments VI–VIII, sometimes absent from segment VI, pores with 7 loculi in the outer ring.

***Dorsum*.** Anal ring on dorsal surface separated from posterior body margin by approximately one-half diameter of ring, 40–53 μm in diameter; with oval cells, some cells bearing spicules; typically with three pairs of setae, although one specimen in type series (JSL090724-05E) has four pairs, each 40–50 μm long. Posterior pair of dorsal ostioles present, diameter of orifice measured along longitudinal axis approximately 40 μm; anterior pair present but barely perceptible except under SEM, reduced compared to posterior pair and poorly developed, diameter of orifice approximately 15 μm. Multilocular disc pores absent.

#### Informal synonyms.

Specimens of *R.montanae* have been previously referred to in the literature as “*Rhizoecus* new sp.” (Schneider and LaPolla 2011). At the time, *R.campensis* and *R.montanae* were thought to comprise a single species associated with *A.dubitata* and the generic assignment was uncertain.

#### Etymology.

The species epithet is derived from the Latin adjective *montanus* (of mountains), indicating that the type series was collected from a mountainous area of Hispaniola.

#### Diagnosis.

*Ripersiellamontanae* sp. nov. is similar in appearance to *R.campensis* sp. nov. The diagnosis section under *R.campensis* explains how the two species may be distinguished. *Ripersiellamontanae* is also similar in appearance to *R.andensis* but the two species can be distinguished as follows (character states for *R.andensis* are given in parentheses): having antennal segments II–IV differing in length (subequal in length); having a comparatively short terminal antennal segment, 40 μm long (comparatively long, 53 μm); having hind legs approximately 275 μm long (240 μm); having the hind trochanter+femur shorter than the tibia+tarsus (the reverse); and having anal lobe setae as long as 59 μm (as long as 33 μm).

#### Comments.

*Ripersiellamontanae* was discovered from two nests of *A.dubitata* (nests DR1–2). The nests were located under stones in a coffee plantation within a few meters of one another. We verified direct species-to-species association (trophobiosis) between the scale insects and ants through observation of attendance by worker ants and by the fact that both colonies contained numerous individuals of the same root mealybug species within their nest chambers and no additional species of scale insects were present. This species was only discovered at high elevation (>1000 m) in the mountainous region of western Dominican Republic near the border with Haiti. Several nests of *A.dubitata* were collected throughout the lowland regions (112–290 m) of Dominican Republic but these nests contained a different associated root mealybug species, *R.campensis*.

### ﻿ Ripersiella pediandensis

Taxon classificationAnimaliaHemipteraRhizoecidae

Schneider & LaPolla sp. nov.

55DC4665-8F50-5901-99D6-AB9EE246BD00

https://zoobank.org/00D70A87-8894-4D9B-BDF2-B79BD397D643

Figure 9

#### Material examined.

***Holotype*.** Peru • 1 adult ♀; Madre de Dios, Dept. Cusco, Cosnipata Valley, Carretera a Manu; -13.0685, -71.5539; 3.viii.2012; TR Schultz leg. (TRS120803-05); collected from *Acropygagoeldii* (group) colony; host not recorded; USNM (nest TRS1: prep S0092B). ***Paratypes*.** Peru • 1 adult ♀; same data as holotype; USNM (nest TRS1: prep S0092A) • 1 adult ♀; same data as holotype; UNAB (nest TRS1: prep TRS120803-05A).

#### Description.

Adult female (*N* = 3). Appearance in life not recorded.

**General.** Mounted on microscope slide, body membranous, broadly oval in young adults to nearly circular in more mature specimens, 1.31–1.53 (1.43) mm long, widest near abdominal segments III–V, 1.01–1.44 (1.21) mm wide. Abdomen smoothly rounded; abdominal segment VIII 258–275 (258) μm wide at base. Anal lobes poorly developed with several flagellate setae on venter and dorsum, ranging from 10–40 μm long, each with group of 3 distinctly stouter setae situated near dorsal margin 71–108 μm long. Body setae flagellate, 11–33 μm on head, 11–15 μm on thorax, 10–40 μm on abdominal segments. Trilocular pores abundant and distributed among body setae; bitubular cerores absent. Microtrichia present; presence of rounded dermal micro-bumps uncertain. Eyes absent.

**Figure 9.**
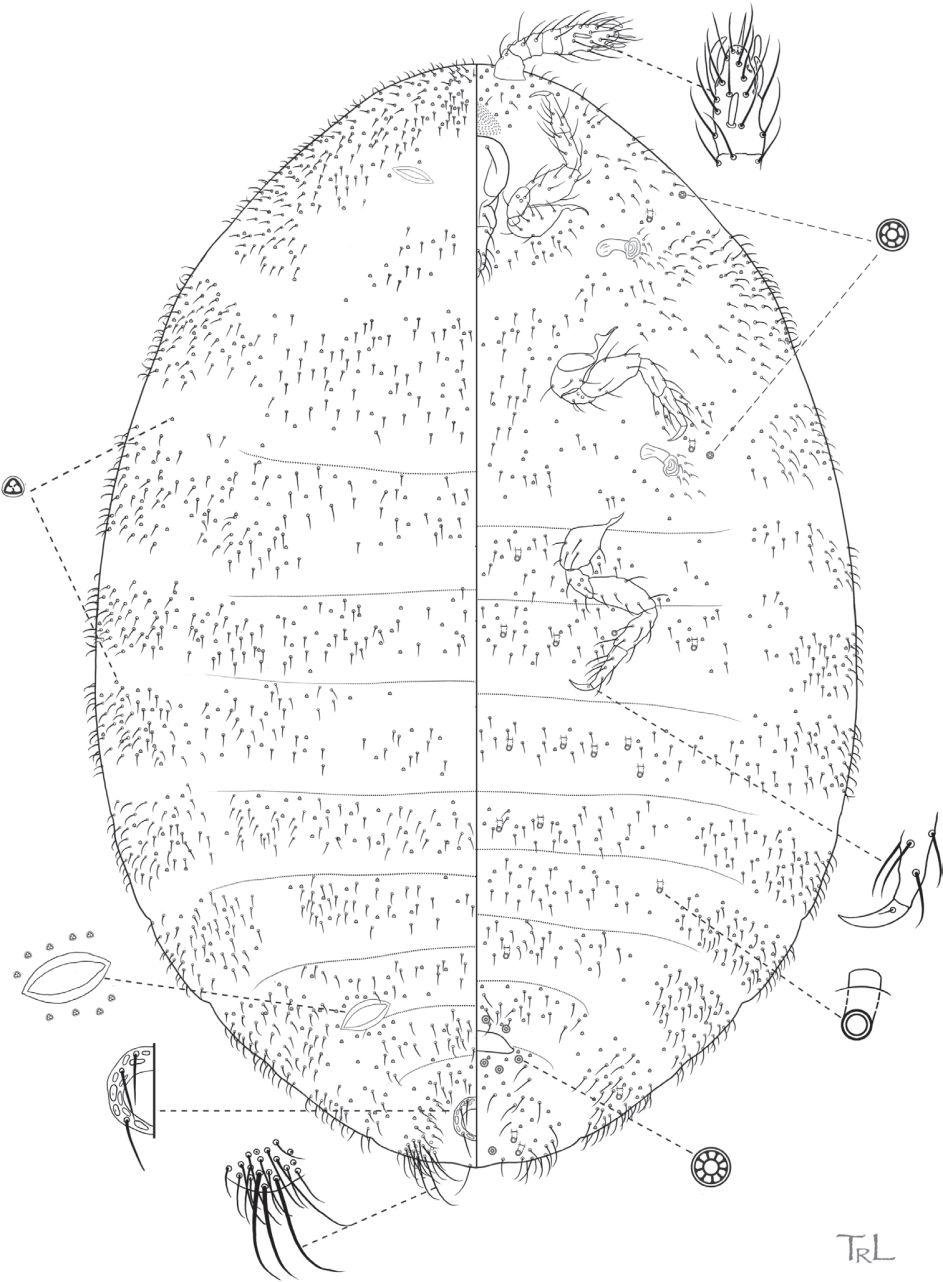
*Ripersiellapediandensis* Schneider & LaPolla sp. nov. Adult female, full body view, illustrated from holotype by T. Litwak (USDA ARS SEL), with edits by SAS.

***Venter*.** Cephalic plate present, with few setae on the plate and several setae and trilocular pores surrounding the ventral and lateral margins. Labium with 3 segments; 92 μm long and 49 μm at widest point. Antennae geniculate, 5-segmented, closely situated near midline on ventral submargin of head; overall length 188–201 (192) μm; length of segment I: 45–50 (48) μm; segment II: 19–24 (19) μm; segment III: 19–21 (19) μm; segment IV: 19–23 (23) μm; segment V: 79–88 (79) μm; apical antennal segment with 1 spine-like and 4 falcate stout sensory setae; a few flagellate setae on each antennal segment, 30–45 μm long; sensorium present on second antennal segment. Legs well developed; overall length of hind leg 346–381 (346) μm; length of hind coxa 42–63 (42) μm; hind trochanter + femur 132–137 (132) μm; hind tibia + tarsus 136–144 (136) μm; hind claw 36–37 (36) μm; each claw with short setose digitule 7.5 μm long; flagellate setae present on each segment, about 25 μm long; 3 stout spine-like setae on inner margin of tibia and tarsus. Circulus absent. Multilocular disc pores present near the vulva on abdominal segments VII–VIII with 9 loculi in the outer ring; near each spiracle a multilocular disc pore with 6 or 7 loculi present. Oral collar tubular ducts present in singular rows or sparsely scattered on median to submedian areas of ventral abdominal segments, 1 or 2 present on thoracic segments near each spiracle.

***Dorsum*.** Anal ring on dorsal surface separated from posterior body margin by approximately 1× diameter of ring, 66–73 μm in diameter; with oval cells, some cells bearing spicules; with three pairs of setae 30–42 μm long. Posterior pair of dorsal ostioles present, diameter of orifice measured along longitudinal axis approximately 44 μm; anterior pair present but smaller than posterior pair, diameter of orifice approximately 29 μm. Multilocular disc pores absent. Oral collar tubular ducts absent.

#### Etymology.

The species epithet is an adjective meaning “from the foot of the Andes”, which combines the noun *pedis* (foot), the adjective *andinus* (pertaining to the Andes Mountains), and suffix -*ensis* (of or from a place).

#### Diagnosis.

*Ripersiellapediandensis* sp. nov. is similar in appearance to *R.andensis* but the two species can be distinguished as follows (character states for *R.andensis* are given in parentheses): having oral collar tubular ducts present on the venter (absent); having comparatively long claws, 37 μm (comparatively short, 24 μm); having comparatively long anal lobe setae, 71–108 μm (comparatively short, 33 μm). The body and appendages of *R.pediandensis* are also longer in comparison. Compared to *R.kelloggi* (character states in parentheses), the legs of *R.pediandensis* are larger in proportion to the body (smaller in proportion), the cephalic plate is present (absent), multilocular disc pores are present near the vulva (absent), it is lacking a circulus (bears 2 small circuli), and the longest anal lobe setae are 71–108 μm long (less than 30 μm long). See the diagnosis under *R.telalia* sp. nov. for a comparison to that species.

#### Comments.

The association between *R.pediandensis* and a species of *Acropyga* (within the *goeldii* group) is lacking information on observations that were made to confirm direct trophobiosis between these partners, and only a single nest was collected. Thus, as in some other cases discussed by Schneider and LaPolla (2020), we consider this relationship to be speculative (however likely) until it can be confirmed through further collections and observations.

### ﻿ Ripersiella telalia

Taxon classificationAnimaliaHemipteraRhizoecidae

Schneider sp. nov.

4DF4D8D2-402C-5D4A-854B-75F6A9EBA4C7

https://zoobank.org/9BF4827C-F919-49E2-8BBC-B76E3B87B0EA

Figures 10 , 11

#### Material examined.

***Holotype*.** Peru • 1 adult ♀; Madre de Dios, Manu National Park, Cocha Cashu Biological Station, trail intersection of 1:306 and 5A; -11.8833, -71.4000; 10.vi.2019; JS LaPolla, SA Schneider leg.; from large *Acropyga* (possibly) *decedens* nest; USNM (nest PER24-01: prep S0425D). ***Paratypes*.** Peru • 3 adult ♀♀; same data as holotype; USNM (nest PER24-01: preps S0425A,C,F) • 1 adult ♀; same data as holotype; UNMSM (nest PER24-01: prep S0425B) • 1 adult ♀; same data as holotype; UNAB (nest PER24-01: prep S0425E) • 1 adult ♀; same data as holotype; UKNMH (nest PER24-01: prep S0425G).

**Figure 10.**
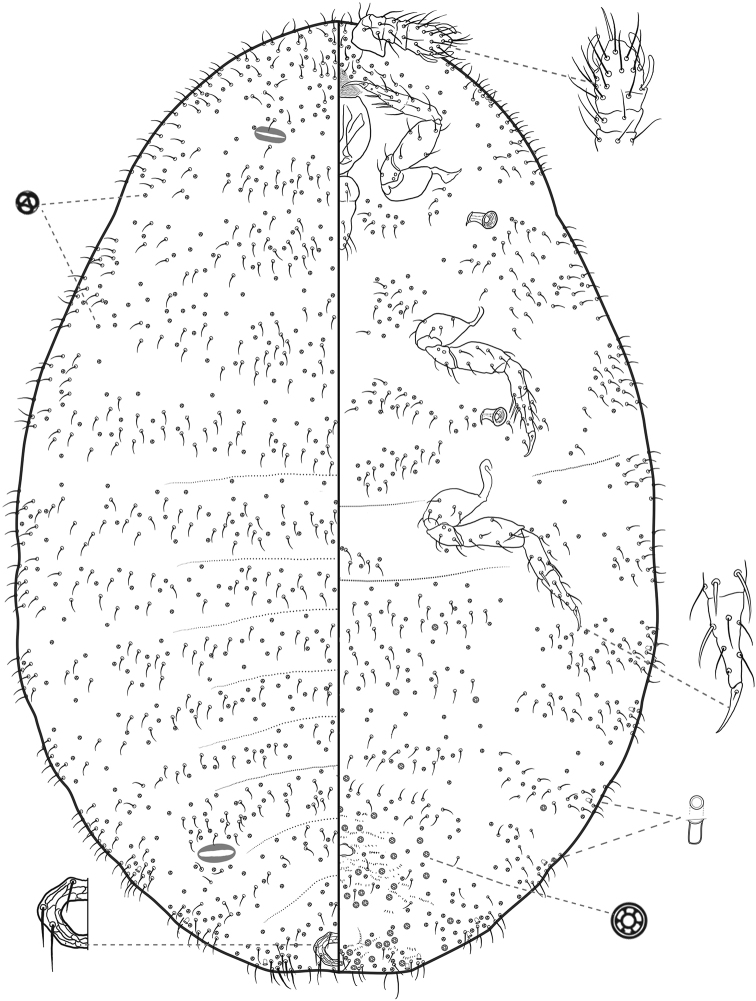
*Ripersiellatelalia* Schneider sp. nov. Adult female, full body view, illustrated from holotype by T. Litwak (USDA ARS SEL), with edits by SAS.

#### Description.

**Figure 11.**
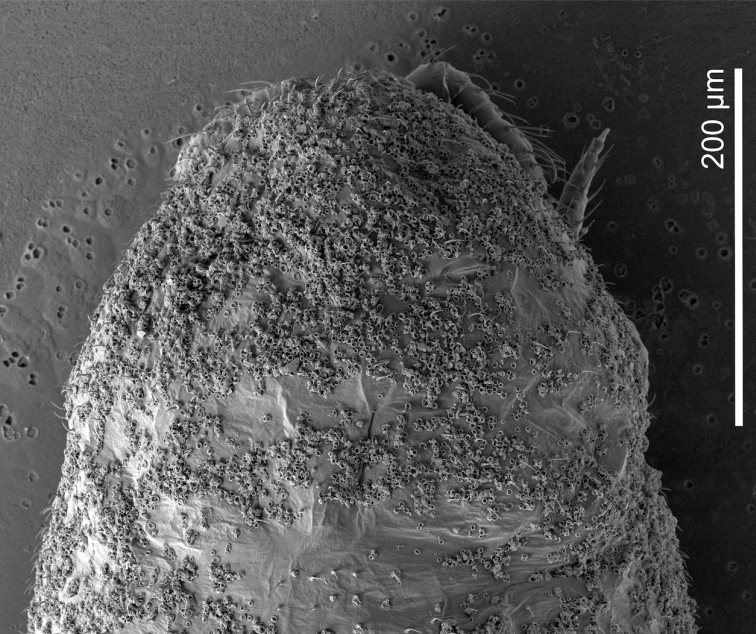
*Ripersiellatelalia* Schneider sp. nov. Scanning electron micrograph (SEM) by J. Mowery (USDA ARS SEL). Dorsal anterior view of adult female head and thorax, showing the presence of anterior ostioles, relatively heavy coating of curled wax deposits, and apparent absence of domed dermal micro-bumps in intersegmental areas.

Adult female (*N* = 7). In life, body bright white to cream colored and visibly coated in powdery white wax (Fig. 11).

**General.** Mounted on microscope slide, body broadly oval and membranous, 0.85–1.07 (0.95) mm long, widest near abdominal segment III, 0.55–0.72 (0.65) mm wide. Abdomen smoothly tapering toward posterior end; abdominal segment VIII about 250 μm wide at base. Anal lobes poorly developed with several stout flagellate setae on venter and dorsum, 16–25 μm long, with longest setae on margin; lacking differentiated set of 3 longer stout setae common to the group. Body setae short and flagellate, 9–12 μm on head, 8–12 μm on thorax, 9–19 μm on abdominal segments. Trilocular pores abundant and distributed among body setae; bitubular cerores absent; 1–4 oral collar tubular ducts present on margins of each abdominal segment VI–VIII and extending as far anterior as III on some specimens. Microtrichia present; rounded dermal micro-bumps apparently absent. Eyes absent.

***Venter*.** Cephalic plate present. Labium with 3 segments; 76 μm long and 39 μm at widest point. Antennae geniculate, 5-segmented, closely situated near midline on ventral submargin of head; overall length about 126–129 (128) μm; length of segment I: 32–35 (32) μm; segment II: 13–16 (16) μm; segment III: 14–17 (15) μm; segment IV: 12–14 (13) μm; segment V: 50–52 (52) μm; on some specimens apical antennal segment retains partial intersegmental line marking obsolete segment VI; with 1 spine-like and 3 falcate stout sensory setae on apical segment; a few flagellate setae on each antennal segment, 15–30 μm long; sensorium present on second antennal segment. Legs well developed; overall length of hind leg 267–284 (270) μm; length of hind coxa 41–52 (41) μm; hind trochanter + femur 101–105 (102) μm; hind tibia + tarsus 97–101 (101) μm; hind claw 26–30 (28) μm; each claw with short setose digitule 2–3 μm long; flagellate setae present on each segment, around 13–20 μm long; 3 stout spine-like setae on inner margin of tibia and tarsus. Circulus absent. Multilocular disc pores in irregular rows or groups on median to submedian of III–VIII, sparse on III–VI, abundant on segments VII and VIII with some located on submargins, pores with 6 or 7 loculi in the outer ring.

***Dorsum*.** Anal ring near dorsal margin, 41–46 μm in diameter; with oval cells, some cells bearing spicules; with 3 pairs of setae 26 μm long. Posterior pair of dorsal ostioles present, diameter of orifice measured along longitudinal axis approximately 42 μm; anterior pair present and slightly smaller than posterior pair, diameter of orifice approximately 33 μm. Multilocular disc pores absent.

#### Informal synonyms.

Specimens of *R.telalia* have been previously referred to in the literature as “Ripersiellanearandensis (ii)” (Schneider et al. 2022).

#### Etymology.

The species epithet is a genitive noun meaning “distant conversation”, combining the Greek noun *lalia* (conversation, talk) with the suffix *tele* (at a distance). Throughout the isolation of the modern pandemic, quarantine chat groups helped us maintain a much-needed sense of community. This species is named in tribute to these groups, and is specifically dedicated to Josh, Justin, and Mark. The name can be considered a double entendre, as it also alludes to the species’ symbiosis with ants as an ongoing conversation between distantly related partners.

#### Diagnosis.

*Ripersiellatelalia* sp. nov. is similar to *R.pediandensis* sp. nov. in possessing oral collar tubular ducts, but the distribution of ducts differs between species. In *R.telalia* these ducts are restricted to the margins of abdominal segments with only 1–4 present per segment, whereas in *R.pediandensis* they are present in ventral bands often exceeding four per segment. The anterior pair of dorsal ostioles are large and more obviously developed in *R.telalia* relative to the other species discussed in this work.

#### Comments.

*Ripersiellatelalia* was discovered from quite a large nest of *Acropyga* (probably) *decedens*. We estimated in the field that the nest comprised thousands of individual ants and scales. Their direct association was confirmed through observation of the colony using a nest-box, as described by Schneider et al. (2022). After specimens were collected into a nest-box, worker ants gathered trophobionts into a protective cluster and were actively engaged in attending to them.

### ﻿Key to the Neotropical species of *Ripersiella* lacking bitubular cerores

In Szita et al. (2020), the first couplet in their key to adult females from the Neotropical region distinguishes between species with and species without bitubular cerores (bitubular ducts). Our key to adult females below, restricted to the species lacking bitubular cerores, would substitute their couplets 18–19.

**Table:** 

1	Ventral multilocular disc pores absent	**2**
–	Ventral multilocular disc pores present	**4**
2	Oral collar tubular ducts present; abdomen bearing 2 circuli	***R.kelloggi* Ehrhorn & Cockerell**
–	Oral collar tubular ducts absent; abdomen bearing 1 circulus	**3**
3	Antennae 5-segmented, about 180 μm long; metathoracic legs about 350 μm long; anal lobe setae short, 18–28 μm	***R.colombiensis* (Hambleton)**
–	Antennae 6-segmented, about 135 μm long; metathoracic legs about 285 μm long; anal lobe setae long, 57–60 μm	***R.illicians* Schneider & LaPolla sp. nov.**
4	Oral collar tubular ducts absent	**5**
–	Oral collar tubular ducts present	**7**
5	Antennal segment V shorter than combined length of segments II–IV; antennal segments II–IV differing in length	***R.montanae* Schneider & LaPolla sp. nov.**
–	Antennal segment V equal to or exceeding combined length of segments II–IV; antennal segments II–IV subequal in length	**6**
6	Ventral multilocular disc pores present on abdominal segments III, IV, or V; longest anal lobe setae distinctly longer than body setae, up to 75 μm long	***R.campensis* Schneider & LaPolla sp. nov.**
–	Ventral multilocular disc pores always absent from segments III–IV and rarely on V; longest anal lobe setae similar in length to body setae, up to 35 μm long	***R.andensis* (Hambleton)**
7	Oral collar tubular ducts present on medial and submedial areas of abdominal segments; longest anal lobe setae distinctly longer than body setae	***R.pediandensis* Schneider & LaPolla sp. nov.**
–	Oral collar tubular ducts restricted to submarginal and marginal areas of abdominal segments; longest anal lobe setae similar in length to body setae	***R.telalia* Schneider sp. nov.**

## ﻿Discussion

With the inclusion of several new species in this complex, we can begin to recognize shared traits that may relate to their intimate association with *Acropyga* ants. Structures relating to wax production are of interest. Species in the *andensis*-complex are all conspicuously lacking tubular cerores, which is an uncommon trait among the Rhizoecidae (Kozár and Konczné Benedicty 2007). Loss and reduction of wax-producing structures is suspected to coincide with ant-association among sternorrhynchous insects (Way 1963; Delabie 2001; Ivens 2015); for example, the Xenococcidae have no wax pores, with one unusual exception (Williams 2004). Along these lines, multiple (though not all) species in the *andensis*-complex seem to produce little to no wax from their trilocular pores. Certain species were at first thought to be free of wax until closer inspection under SEM showed that wax deposits are indeed present (Figs 3, 8) but only apparent under high magnification. Why some species produce visible amounts of wax (e.g., *R.illicians* and *R.telalia*) and others do not (e.g., *R.campensis* and *R.montanae*) may relate to abiotic conditions within the nest or perhaps to the duration of their lineage’s association with *Acropyga* ants, assuming the loss of wax production occurs gradually over generations.

The trend toward reduction of ostioles among the *andensis*-complex appears to be related to ant association as well. The anterior pair of dorsal ostioles is reduced in size compared to the posterior pair or they are lost entirely among species in the complex. We also note a gradient in the degree of ostiole development among species, ranging from *R.telalia* (Figs 10, 11) and *R.pediandensis* (Fig. 9) with the most prominent pairs at 33 μm and 29 μm in diameter, respectively, down to two species (*R.colombiensis* and *R.illicians*) who have lost them entirely. *Ripersiellacampensis* and *R.montanae* have anterior ostioles reduced in size (10–15 μm in diameter) and poorly developed to the point that they are essentially undetectable when viewed under a light microscope; we only recognized their presence due to SEM imaging (Fig. 3). Ostioles are missing entirely among other scale groups associated with *Acropyga* including the Xenococcidae and some other species of Rhizoecidae (Tanaka 2016; Schneider and LaPolla 2020), further suggesting that reduction of ostioles among the *andensis*-complex is due to their relationship with ants. Ostioles are likely involved in predator defense (discussed in detail by Williams 1978), and these root mealybugs have outsourced their defense against natural enemies to their mutualist partner. Furthermore, reduction in ostiole size among mealybugs is typically correlated with increased dorsal wax production or the production of a felted ovisac covering the body, as in *Antonina* Signoret (Williams 1978); species in the *andensis*-complex produce little to no wax at all. Interestingly, mealybugs from the tribe Allomyrmococcini Williams (Hemiptera, Pseudococcidae), the obligate associates of herdsmen ants (Dill et al. 2002), have trended in the opposite direction and possess dramatically enlarged ostioles that may exude ant attractants (Williams 1978). Therefore, the degree of ostiole development in either direction, whether becoming enlarged or reduced, apparently correlates to a close ecological relationship with ants.

Finally, the “hairiness” of species seems potentially important among groups of trophobiotic mealybugs. For example, members of Xenococcidae tend to be densely covered in setae and/or microtrichia (Williams 1998, 2004; Schneider and LaPolla 2011), as are species of Allomyrmococcini. Williams (1978) suggested that a dense covering of setae may trap a layer of air and act as an alternative to waterproofing in the absence of wax production. Microtrichia can similarly provide waterproofing (Neumann and Woermann 2009). Anecdotally, we note that certain species in the *andensis*-complex appear slightly more densely covered in setae than is typical (e.g., *R.montanae* and *R.pediandensis*). However, further study is required to determine if the relative densities of setae or microtrichia significantly differ among ant-associated rhizoecids compared to those that are free-living. Their setae are clearly less densely distributed than trophobionts from other groups, like the Xenococcidae and Allomyrmococcini. High-resolution SEM images of *R.campensis*, *R.illicians*, and *R.montanae* (Figs 3, 6, 8) captured an abundance of domed dermal micro-bumps, similar to microtrichia, which are concentrated in intersegmental regions and the attachment points of appendages and are not visible under light microscopy. This feature could be typical within the family, which we will only discover through further sampling and imaging of free-living and ant-associated species. Such dermal micro-bumps are not apparent on *R.telalia*, which coincidentally has a relatively dense coating of wax (Fig. 11) compared to the other species and the largest anterior ostioles. Determining the identity and function of these dermal micro-bumps and their correlation to waxiness and ant association offers interesting directions for future research.

## Supplementary Material

XML Treatment for Ripersiella

XML Treatment for Ripersiella andensis

XML Treatment for Ripersiella campensis

XML Treatment for Ripersiella colombiensis

XML Treatment for Ripersiella illicians

XML Treatment for Ripersiella montanae

XML Treatment for Ripersiella pediandensis

XML Treatment for Ripersiella telalia
